# Effects of High-Flow Nasal Cannula and Helmet Continuous Positive Airway Pressure in Acute Hypoxemic Respiratory Failure

**DOI:** 10.1097/CCM.0000000000007116

**Published:** 2026-04-01

**Authors:** Silvia Coppola, Mariarosa Pelliccia, Tommaso Pozzi, Giulia Catozzi, Cosmo Rocco, Alessandro Monte, Geraldina Besana, Davide Chiumello

**Affiliations:** 1 Department of Anesthesia and Intensive Care, ASST Santi Paolo e Carlo, San Paolo University Hospital Milan, Milan, Italy.; 2 Department of Health Sciences, University of Milan, Milan, Italy.; 3 Coordinated Research Center on Respiratory Failure, University of Milan, Milan, Italy.

**Keywords:** acute hypoxemic respiratory failure, acute respiratory distress syndrome, continuous positive airway pressure, high-flow nasal cannula, respiratory support

## Abstract

**HEADINGS:**

The effects of high-flow nasal cannula (HFNC) and continuous positive airway pressure (CPAP) in patients with acute hypoxemic respiratory failure (AHRF) on respiratory mechanics and inspiratory efforts are not entirely understood.

**OBJECTIVES:**

To compare the physiologic effects of HFNC and helmet CPAP with respect to conventional oxygen therapy (COT) in terms of respiratory mechanics, inspiratory effort, gas exchange, and hemodynamics during AHRF.

**DESIGN:**

Crossover study.

**SETTING:**

General surgical-medical ICU of San Paolo University Hospital, Milan, Italy.

**PATIENTS:**

Thirty-three adult patients with AHRF, defined as an Pao_2_ less than 60 mm Hg or an Pao_2_/Fio_2_ less than 300 with a positive end-expiratory pressure (PEEP) level greater than or equal to 5 cm H_2_O, along with an Paco_2_ less than 45 mm Hg.

**INTERVENTIONS:**

After support with COT, three types of respiratory support were applied in random order: HFNC with 60 L/min of flow and helmet CPAP with 5 or 10 cm H_2_O of PEEP.

**MEASUREMENTS AND MAIN RESULTS:**

Tidal volume, respiratory rate, inspiratory esophageal (ΔPes), and airway pressure swings were measured and an arterial blood gas analysis, along with hemodynamic data, was obtained after 20 minutes from the application of each respiratory support device. The application of HFNC and helmet CPAP at both 5 and 10 cm H_2_O of PEEP reduced minute ventilation (9.2 ± 3.2, 8.8 ± 2.3, and 9.3 ± 2.7 vs. 10.9 ± 3.3 L/min; *p* < 0.001) and ΔPes (–6.0 cm H_2_O [–7.8 to –4.0 cm H_2_O], –5.8 cm H_2_O [–7.2 to –4.5 cm H_2_O], and –5.9 cm H_2_O [–8.0 to –4.0 cm H_2_O] vs. –7.5 cm H_2_O [–10.8 to –6.5 cm H_2_O]; *p* < 0.001) while increasing Pao_2_/Fio_2_ (188 ± 57, 208 ± 62, and 213 ± 69 vs. 129 ± 32; *p* < 0.001) with respect to COT; the application of 10 cm H_2_O of PEEP with helmet CPAP did not reduce inspiratory effort indices or increased oxygenation, but worsened mechanical power compared with HFNC and helmet CPAP with 5 cm H_2_O of PEEP.

**CONCLUSIONS:**

In patients with mild and moderate AHRF, HFNC and helmet CPAP ameliorated minute ventilation and respiratory rate, reduced inspiratory effort, and increased oxygenation compared with COT; the application of 10 cm H_2_O of PEEP during CPAP support worsened mechanical power.

**KEY POINTS**

**Question**: This study evaluated the physiologic effects of conventional oxygen therapy (COT), high-flow nasal cannula (HFNC), and helmet continuous positive airway pressure (CPAP) with 5 and 10 cm H_2_O during acute hypoxemic respiratory failure (AHRF).

**Findings**: This crossover study demonstrated that HFNC and helmet CPAP reduced minute ventilation, ameliorated inspiratory effort indices, and increased oxygenation with respect to COT; the application of 10 cm H_2_O of positive end-expiratory pressure (PEEP) did not provide benefits, increasing mechanical power.

**Meaning**: HFNC and helmet CPAP could be useful during AHRF, thanks to their effect on lung mechanics, inspiratory effort, and oxygenation. However, the application of high levels of PEEP could increase the mechanical power without additional physiologic benefits.

Patients with acute hypoxemic respiratory failure (AHRF) are characterized by an impairment in gas exchange and an increase in the work of breathing (1, 2). In patients with AHRF, the most used noninvasive respiratory support devices are high-flow nasal cannula (HFNC) and continuous positive airway pressure (CPAP) (3–6). Due to their ease of use, HFNC and CPAP can be applied also outside the ICU, allowing a more extensive application of noninvasive respiratory support in a broader number of patients. In particular, helmet CPAP has been extensively applied for long-term treatments with high level of positive end-expiratory pressure (PEEP) (4, 5, 7).

The application of PEEP improves ventilation/perfusion distribution, increases oxygenation, and end-expiratory lung gas volume (EELV), possibly reducing the work of breathing (8–10). Alternatively, HFNC has been proposed in patients with mild-moderate AHRF (6). HFNC is able to deliver a flow of humidified oxygen up to 50–60 L/min, through a dedicated nasal interface; it has been demonstrated that HFNC could promote an increase in the EELV by generating a small level of PEEP, with a consequent improvement in oxygenation, along with a better Co_2_ clearance and the reduction of inspiratory effort due to anatomical dead space washout (11–17).

In a crossover randomized study comparing HFNC, CPAP, and noninvasive ventilation (NIV) in moderate-severe hypoxemic respiratory failure, CPAP, and HFNC showed a similar effect on inspiratory effort and respiratory rate (8). However, it should be noted that an average PEEP level of 13 cm H_2_O was applied with helmet CPAP, which is an unusual high level with possible negative effects such as on the long-term tolerance of the device and overstress of the lung (8, 18). Recently, Tuffet et al (19) compared the effects of low levels of CPAP via an oronasal mask and HFNC in terms of oxygenation and distribution of ventilation in patients with AHRF, showing that HFNC was associated with lower minute ventilation and arterial Co_2_, but also with lower oxygenation.

Furthermore, the beneficial effects of HFNC and CPAP are mainly related to avoiding the intubation (7). However, the maintenance of spontaneous breathing with a high minute ventilation and inspiratory effort increases the risk of patient self-inflicted lung injury (P-SILI) (20–23). During CPAP and NIV, the assessment of inspiratory effort using esophageal pressure (Pes) has been shown to accurately predict the risk of failure (18, 24). The effects of HFNC and CPAP in patients with AHRF on minute ventilation and inspiratory efforts are not well understood. Two studies conducted in patients with AHRF with and without COVID-19, reported that HFNC and CPAP demonstrated similar inspiratory effort and tidal volume (25, 26).

The aim of this crossover study was to assess and compare the physiologic effects of conventional oxygen therapy (COT), HFNC, and helmet CPAP at a low (5 cm H_2_O) and high (10 cm H_2_O) of PEEP in terms of respiratory mechanics, indices of inspiratory effort, mechanical power (MP), gas exchange, and hemodynamics in patients with AHRF.

## MATERIALS AND METHODS

### Study Population

This was a crossover study performed from October 2023 to February 2024 at the ICU of the ASST Santi Paolo Carlo, San Paolo University Hospital, Milan, Italy. All consecutive adult patients who presented AHRF, defined as a Pao_2_ less than 60 mm Hg or a Pao_2_/Fio_2_ less than 300 with a PEEP level greater than or equal to 5 cm H_2_O, along with a Paco_2_ less than 45 mm Hg and the need for noninvasive respiratory support without any prior attempt at respiratory support, were included. Exclusion criteria were hemodynamic instability, altered mental status (defined as a Glasgow Coma Scale < 9), indication for immediate intubation, and contraindication to esophageal balloon placement. The study was approved by the institutional review board of the ASST Santi Paolo e Carlo (protocol number 217-204, study name: “HFNC CPAP”) on September 2023; informed consent was obtained according to the Italian regulations; and procedures were followed in accordance with the ethical standards of the responsible committee on human experimentation (institutional or regional) and with the Helsinki Declaration of 1975.

### Study Protocol

Patients were enrolled within the first 48 hours from ICU admission. Upon enrollment, patients were equipped with an esophageal balloon catheter (NutriVent, Sidam, Mirandola, Italy) to measure Pes (27) and a custom-made nasal pressure monitoring system, placed and sealed at one nostril to assess airway pressure (Paw) (28). The esophageal balloon catheter, consisting of a 108 cm long tube with a 10 cm long walled balloon, was positioned in the lower section of the esophagus, to reach a depth of between 45 and 55 cm measured from the mouth. Subsequently, the balloon was inflated with 4 mL of air (27). The amount of gas in the balloon was periodically checked throughout the experiment. See **Supplementary Digital Content** (https://links.lww.com/CCM/H926) for additional methods about esophageal balloon catheter positioning.

Initially, patients were supported with COT by a Venturi mask, with a Fio_2_ to maintain a peripheral oxygen saturation greater than 80%. After the measurements, using a randomized temporal sequence generated by a block algorithm, patients underwent three observations timepoints: HFNC and helmet CPAP at 5 and 10 cm H_2_O of PEEP. Randomization was conducted prior to noninvasive respiratory support initiation.

HFNC was delivered via dedicated nasal cannulas with a gas flow of 60 L/min through a heated humidifier (AIRVO 2; Fisher & Paykel Healthcare, Auckland, New Zealand); this flow rate was chosen in order to optimize the effects on the inspiratory effort and lung mechanics (12). CPAP was administered via a transparent plastic helmet of the appropriate size with a gas flow of 60 L/min supplied by a high-flow generator provided with an adjustable PEEP valve (VitalSigns, Totowa, NJ). A heated humidifier was applied (Hidraltis, 9500FM; Burke & Burke, Assago, Italy). Fio_2_ was maintained unmodified with respect to COT timepoint.

If at any point worsening of the respiratory failure, hemodynamic instability, or need for immediate intubation occurred, the study was terminated and the patient was excluded. At the end of the study protocol, respiratory support was set according to the attending clinician.

### Data Collection and Analysis

Demographics and clinical characteristics of patients were recorded upon enrollment. An arterial blood gas analysis, along with respiratory and hemodynamic data were collected after 20 minutes of application of each noninvasive ventilatory support. Tidal volume and respiratory rate were measured by a noninvasive system, which assesses thoracic impedance variations with a padset sensor (ExSpiron 1Xi system; Respiratory Motion, Inspired Innovation, Watertown, MA) (29). See Supplementary Digital Content (https://links.lww.com/CCM/H926) for additional methods about noninvasive tidal volume and respiratory rate monitoring. Both Pes and Paw were monitored continuously by the OptiVent monitoring system (OptiVent; Sidam). All variables were recorded as the average value of three consecutive measurements within 2 minutes.

### Computed Variables

The following variables were computed: airway driving pressure (ΔPaw) and inspiratory Pes swings (ΔPes) as the difference between end-inspiratory and end-expiratory airway and Pes, respectively. Muscular pressure (Pmus) was calculated as:


Pmus=Prel,cw−ΔPes

where Prel, cw is the chest wall elastic recoil pressure, which can be calculated assuming a theoretical value of chest wall compliance as 4% of the predicted vital capacity, and then using measured tidal volume to obtain chest wall elastic recoil pressure (Prel,cw = chest wall compliance × tidal volume) (30, 31). The dynamic lung elastance (E_L,dyn_) was computed as:


$$E_{L,dyn}={\;}^{\Delta Pes}/{\;}_{VT}$$

where Vt is tidal volume in liters. The MP during spontaneous breathing was calculated as:


$$\begin{array}{l} Mechanical\;Power\left(MP\right)=0.098\times RR\;\\ \times \left[VT^{2}\times \left(0.714\times \frac{\Delta Pes}{VT}+RR\times 0.5\right)+VT\times PEEP\right] \end{array}$$

where 0.098 is the constant to convert 1 cm H_2_O into J/min and 0.714 is a constant that accounts for the ratio between respiratory system and lung elastance assumed to be 0.7, as previously done by Gattarello et al (32) in COVID-19 patients and demonstrating to be associated with higher risk of respiratory failure. The ventilatory ratio (VR) was computed as:


$$VR=\frac{Minute\;Ventilation\times PaCO_{2}}{0.1\times IBW\times 40}$$

where IBW is ideal body weight (33).

### Statistical Analysis

Continuous data are reported as mean ± sd or median (interquartile range), as appropriate; categorical data are reported as number (%). One-way analysis of variance for repeated measures or Friedman test were performed to assess differences within respiratory support settings in the four observation timepoints. A post hoc analysis with Bonferroni correction was performed for paired multiple comparisons. A *p* value of less than 0.05 was considered as statistically significant. Statistical analyses were performed using R Studio (RStudio. Integrated Development for R. RStudio, PBC, Boston, MA).

## RESULTS

A total of 33 patients were enrolled, and no patient had to be excluded from the study. **Table 1** shows the baseline demographic and clinical characteristics of the patients. The median age was 59 years (40–70 yr) with a Simplified Acute Physiology Score II of 26 (19–33). Twenty-seven (82%) had a pulmonary origin of the AHRF and among these patients 24 (89%) presented community-acquired pneumonia. The median Pao_2_/Fio_2_ and Paco_2_ at enrollment were 206 mm Hg (172–232 mm Hg) and 38 mm Hg (35–41 mm Hg), respectively.

**TABLE 1.** Baseline Characteristics of the Study Population

Characteristics	*n* = 33
Age, yr	59 (40–70)
Male sex, *n* (%)	19 (58)
Body mass index, kg/m^2^	27 (25–32)
ICU length of stay, d	4 (3–5)
Time from ICU admission to study day, d	1 (1–2)
Underwent ETI, *n* (%)	5 (15)
Time from ICU admission to ETI, d	0 (0–1)
Sequential Organ Failure Assessment score	3 (2–3)
Acute Physiology and Chronic Health Evaluation II score	7 (3–10)
Simplified Acute Physiology Score II	26 (19–33)
Respiratory failure etiology, *n* (%)	
Pulmonary	27 (82)
Community-acquired pneumonia	24 (88)
Hospital-acquired pneumonia	2 (8)
Other	1 (4)
Extrapulmonary	6 (18)
Sepsis	3 (49)
Trauma	1 (17)
Pancreatitis	1 (17)
Other	1 (17)

At Positive End-Expiratory Pressure = 5 cm H_2_O	
Arterial pH	7.45 (7.41–7.46)
Pao_2_, mm Hg	88 (72–105)
Pao_2_/Fio_2_	206 (172–232)
Oxygenation impairment severity, *n* (%)	
Mild	18 (55)
Moderate	14 (42)
Severe	1 (3)
Paco_2_, mm Hg	38 (35–41)
Bicarbonates, mEq/L	25 (23–27)
Hemoglobin, g/dL	11.9 (10.8–12.6)
Arterial sodium, mEq/L	136 (135–138)
Arterial potassium, mEq/L	3.97 (3.74–4.19)
Lactates, mmol/L	0.99 (0.81–1.68)
28-d mortality, *n* (%)	1 (3)

ETI = endotracheal intubation.

Data are presented as median (interquartile range) or *n* (%).

### Respiratory Parameters and Mechanics

**Figure 1.**
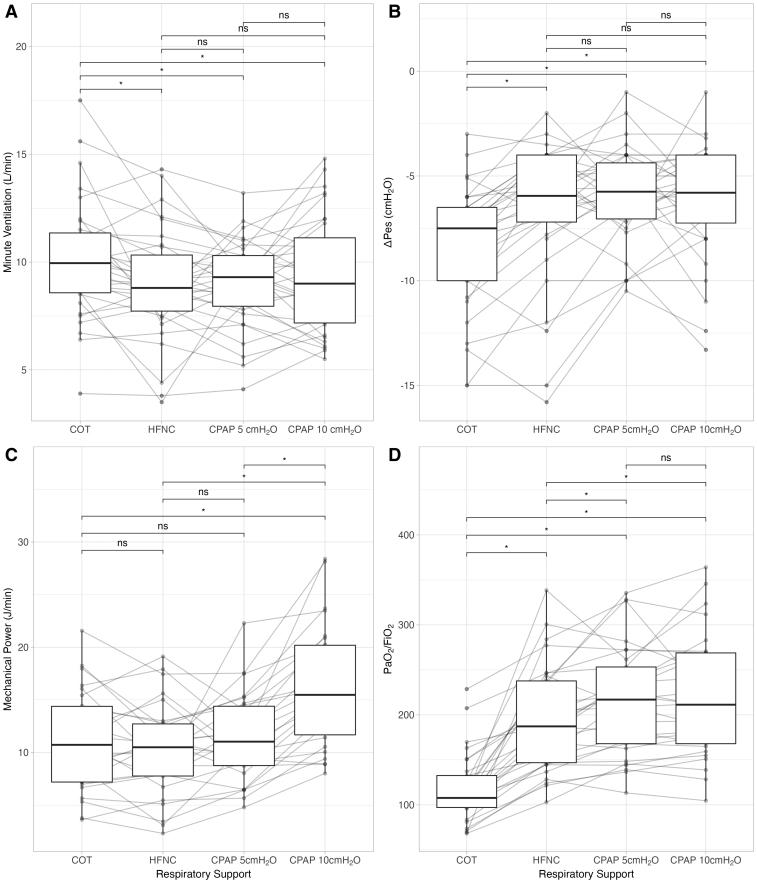
Minute ventilation (**A**), inspiratory esophageal pressure swings (ΔPes) (**B**), mechanical power (**C**), and Pao_2_/Fio_2_ ratio (**D**) according to respiratory supports. *p < 0.050. COT = conventional oxygen therapy, CPAP = continuous positive airway pressure, HFNC = high-flow nasal cannula, ns = not significant.

The comparison within respiratory support devices in terms of respiratory mechanics, inspiratory effort, and gas exchange is presented in **Table 2** (see also **Fig. 1**). When supported with HFNC and helmet CPAP (both with 5 and 10 cm H_2_O of PEEP), patients had a similar respiratory rate and tidal volume. The minute ventilation was significantly lower with HFNC and helmet CPAP compared with COT with facial mask. With HFNC, respiratory rate decreased with respect to COT (19 ± 6 vs. 22 ± 5 beats/min; *p* < 0.050), while tidal volume was similar; specularly, during helmet CPAP both with 5 and 10 cm H_2_O of PEEP tidal volume decreased (450 ± 120 and 450 ± 120 vs. 500 ± 150 mL; *p* < 0.050) but respiratory rate was unchanged with respect to COT. Notably, tidal volume during helmet CPAP at both levels of PEEP was significantly lower also with respect to HFNC (*p* < 0.050).

**TABLE 2.** Respiratory Mechanics, Inspiratory Effort, and Gas Exchange During the Four Respiratory Support Devices Application Throughout the Study

*n* = 33	Conventional Oxygen Therapy	High-Flow Nasal Cannula	CPAP (PEEP 5 cm H_2_O)	CPAP (PEEP 10 cm H_2_O)	*p*
Respiratory parameters and mechanics					
Respiratory rate, breaths/min	22 ± 5	19 ± 6^a^	20 ± 5	21 ± 5	**0.007**
Tidal volume, mL	500 ± 150	510 ± 180	450 ± 120^a,b^	450 ± 120^a,b^	**0.003**
Tidal volume per kg predicted body weight, mL/kg	5.9 (5.3–6.9)	5.6 (5.0–7.1)	5.6 (4.4–6.7)	5.6 (4.6–6.8)	**0.078**
Minute ventilation, L/min	10.9 ± 3.3	9.2 ± 3.2^a^	8.8 ± 2.3^a^	9.3 ± 2.7^a^	**< 0.001**
Inspiratory esophageal pressure swings, cm H_2_O	–7.5 (–10.8 to –6.5)	–6.0 (–7.8 to –4.0)^a^	–5.8 (–7.2 to –4.5)^a^	–5.9 (–8.0 to –4.0)^a^	**< 0.001**
Muscular pressure, cm H_2_O	7.7 (6.7–10.6)	6.1 (4.2–7.6)^a^	5.9 (4.4–7.4)^a^	6.1 (4.2–7.7)^a^	**< 0.001**
Airway pressure, cm H_2_O	–4.5 ± 3.3	–4.5 ± 3.1	–3.1 ± 2.1^a,b^	–3.9 ± 2.3^c^	**0.038**
Dynamic lung elastance, cm H_2_O/L	18.1 ± 7.0	14.2 ± 6.5^a^	14.3 ± 6.7^a^	14.3 ± 6.8^a^	**0.002**
Mechanical power, J/min	12.7 ± 7.5	11.9 ± 7.0	12.1 ± 4.7	17.9 ± 7.5^a,b,c^	**< 0.001**
Gas exchange					
Fio_2_, %	40 (40–50)	40 (40–50)	40 (40–50)	40 (40–50)	
Pao_2_, mm Hg	56 (50–71)	77 (66–99)^a^	90 (72–101)^a,b^	91 (76–125)^a,b^	**< 0.001**
Pao_2_/Fio_2_, mm Hg	129 ± 32	188 ± 57^a^	208 ± 62^a,b^	213 ± 69^a,b^	**< 0.001**
Paco_2_, mm Hg	36 (32–39)	36 (35–39)	36 (35–40)	39 (34–41)^a,b^	**0.024**
Ventilatory ratio	2.0 ± 0.7	1.7 ± 0.7^a^	1.7 ± 0.5^a^	1.8 ± 0.6	**0.004**

CPAP = continuous positive airway pressure, PEEP = positive end-expiratory pressure.

a Statistically significant at comparison with conventional oxygen therapy.

b Statistically significant at comparison with high-flow nasal cannula.

c Statistically significant at comparison with CPAP with PEEP 5 cm H_2_O.

A *p* < 0.05 (in bold) was considered as statistically significant.

Data are presented as median (interquartile range) or mean ± sd.

### Inspiratory Effort

When supported with HFNC and helmet CPAP both at 5 and 10 cm H_2_O of PEEP, patients had a significantly lower ΔPes (–6.0 cm H_2_O [–7.8 to –4.0 cm H_2_O], –5.8 cm H_2_O [–7.2 to –4.5 cm H_2_O] and –5.9 cm H_2_O [–8.0 to –4.0 cm H_2_O] vs. –7.5 cm H_2_O [–10.8 to –6.5 cm H_2_O]; respectively; *p* < 0.001) and Pmus (6.1 [4.2–7.6], 5.9 [4.4–7.4], and 6.1 [4.2–7.7] vs. 7.7 [6.7–10.6], respectively; *p* < 0.001) compared with COT, while there were no differences among HFNC and helmet CPAP. The increase in PEEP level during helmet CPAP support did not change both ΔPes and Pmus. The MP did not change within COT, HFNC, and helmet CPAP at 5 cm H_2_O of PEEP. At 10 cm H_2_O of PEEP during helmet CPAP, the MP significantly increased compared with COT, HFNC and CPAP with 5 cm H_2_O of PEEP (17.9 ± 7.5 vs. 12.7 ± 7.5, 11.9 ± 7.0, and 12.1 ± 4.7; respectively; *p* < 0.001) (see Table 2 and Fig. 1).

### Gas Exchange

Helmet CPAP both at 5 and 10 cm H_2_O of PEEP significantly increased oxygenation as compared with both COT and HFNC (208 ± 62 and 213 ± 69 vs. 129 ± 32 vs. 188 ± 57, respectively; *p* < 0.001); the increase of PEEP during CPAP support did not ameliorate oxygenation (208 ± 62 vs. 213 ± 69; *p* > 0.050). During COT, HNFC and helmet CPAP with 5 cm H_2_O of PEEP, patients maintained a similar Paco_2_, while it was significantly higher with helmet CPAP at 10 cm H_2_O of PEEP (36 mm Hg [32–29 mm Hg], 36 mm Hg [35–39 mm Hg], and 36 mm Hg [35–40 mm Hg] vs. 39 mm Hg [34–41 mm Hg]; respectively; *p* = 0.024). The VR was significantly lower during respiratory support with HFNC and helmet CPAP at 5 cm H_2_O with respect to COT (1.7 ± 0.5 and 1.7 ± 0.7 vs. 2.0 ± 0.7; *p* < 0.050) (Table 2 and Fig. 1).

### Hemodynamics

The arterial blood pressure and heart rate was not different across the four respiratory support devices (**Table 3**).

**TABLE 3.** Hemodynamic Variables in the Four Respiratory Supports Settings

*n* = 33	Conventional Oxygen Therapy	High-Flow Nasal Cannula	CPAP (PEEP 5 cm H_2_O)	CPAP (PEEP 10 cm H_2_O)	*p*
Systolic arterial pressure, mm Hg, mean ± sd	136 ± 14	134 ± 15	137 ± 15	136 ± 15	**0.813**
Diastolic arterial pressure, mm Hg, mean ± sd	68 ± 11	67 ± 11	69 ± 10	72 ± 10	**0.100**
Mean arterial pressure, mm Hg, mean ± sd	90 ± 11	90 ± 10	92 ± 10	91 ± 13	**0.275**
Heart rate, beats/min, median (interquartile range)	80 (74–91)	77 (71–103)	76 (69–93)	77 (70–90)	**0.148**

CPAP = continuous positive airway pressure, PEEP = positive end-expiratory pressure.

Statistically significant at comparison with conventional oxygen therapy.

Statistically significant at comparison with high-flow nasal cannula.

Statistically significant at comparison with CPAP with PEEP 5 cm H_2_O.

A *p* < 0.05 (in bold) was considered as statistically significant.

## DISCUSSION

The main results of the present study can be summarized as follow: 1) HFNC and helmet CPAP, independently of the level of PEEP applied, showed similar minute ventilation and indices of inspiratory effort; 2) HFNC and helmet CPAP reduced minute ventilation and indices of inspiratory effort while increasing oxygenation as compared with COT with facial mask; and 3) the application of 10 cm H_2_O of PEEP with helmet CPAP did not ameliorate indices of inspiratory effort or oxygenation, while increasing Paco_2_ and MP as compared with 5 cm H_2_O of PEEP.

AHRF and acute respiratory distress syndrome, a subset of the most severe form of respiratory failure, which accounts for a high number of ICU admission due to hypoxemia and increased respiratory drive, require noninvasive or invasive respiratory support according to the severity (6, 34, 35). The major advantages of noninvasive respiratory support are the preservation of spontaneous breathing, the maintenance of lung, heart, diaphragm physiology, the improvement in gas exchange, and the reduction of the need of endotracheal intubation (36). However, a potential drawback of the application of any noninvasive respiratory support, is that the maintenance of a vigorous inspiratory effort associated to a high stress and transvascular pressure if not detected, can promote lung edema by enhancing P-SILI and worsen a patient’s outcome (20, 35). Several studies evaluating the timing of intubation in patients with AHRF who failed a trial of noninvasive respiratory support, showed that late intubation was associated with a worse outcome (23, 37, 38).

Among noninvasive respiratory supports, the two most used devices are CPAP and HFNC (3, 6, 8, 39). CPAP is usually delivered by a helmet with a PEEP level selected by the physician according to the severity of the patients; the PEEP level is ensured by the application of an external PEEP valve (40). HFNC consists of a high continuous flow of air/oxygen mixture delivered to the patients by dedicated nasal cannulas; the generated PEEP level and the increase in EELV are related to the amount of gas flow delivered to the patient (3, 12, 15, 41).

### Respiratory Parameters and Mechanics

In order to monitor the beneficial effects and to prevent P-SILI during noninvasive respiratory support it has been suggested to assess minute ventilation and inspiratory efforts (20). In patients with AHRF; however, the tidal volume generated by the CPAP was not related to the tidal volume under HFNC (19). Thus, in order to evaluate the physiologic effects on minute ventilation (respiratory rate and tidal volume), previous studies used electrical tomography impedance technology (42); in the current study, a noninvasive respiratory volume monitoring has been sued, which is able to accurately compute respiratory rate and tidal volume (29).

In the present study, the safest setup to deliver helmet CPAP was applied, through a continuous flow ranging between 40 and 50 L/min with an external PEEP valve to avoid Co_2_ rebreathing (43).

The application of HFNC and helmet CPAP were associated with a similar decrease in minute ventilation with respect to COT; HFNC mainly affected respiratory rate, possibly due to its Co_2_ washout-enhancing mechanism with negligible effects on tidal volume (12). On the contrary, the application of higher levels of PEEP with helmet CPAP resulted in lower tidal volumes without affecting respiratory rate, possibly through a recruitment effect. However, in our study, E_L,dyn_ were similar during HFNC and helmet CPAP, indicating a similar effect of both devices on lung mechanics.

### Inspiratory Effort

To assess inspiratory effort during HFNC and CPAP, it has been proposed to compute a composite visual score based on the chest wall excursion and respiratory rate; however, it can be only intermittently used and it is often inaccurate in predicting a possible failure (44). Thus, ΔPes variations and Pmus have been used as surrogates for inspiratory effort (24, 25). Furthermore, the intensity of inspiratory effort within the first hours of noninvasive respiratory support has been demonstrated to accurately predict a patient’s outcome (24).

In a seminal study, Mauri et al (45) showed that HFNC significantly reduced inspiratory effort and increased the EELV in patients with AHRF. In our study, both helmet CPAP and HFNC similarly decreased inspiratory effort compared with COT, probably due to the effect of PEEP in inducing lung recruitment and ameliorating lung elastance, especially during CPAP, with a contribution of increased Co_2_-washout during HFNC.

Similarly, Vargas et al (25), comparing HFNC and CPAP delivered by a ventilator and a face mask, found that the inspiratory effort was reduced to a similar extent. Furthermore, increasing PEEP from 5 to 10 cm H_2_O with helmet CPAP did not reduce indices of inspiratory effort, probably due to the absence of any further lung recruitment and change in respiratory compliance. Bello et al (46) found that the impact of high PEEP compared with low PEEP on inspiratory effort is highly variable and mainly depends on the balance between improvement and worsening of respiratory system compliance. Accordingly, in our study, the increase in PEEP from 5 to 10 cm H_2_O during helmet CPAP did not ameliorate E_L,dyn_, possibly indicating the lack of any further PEEP-induced alveolar recruitment.

In addition to the assessment of the indices of inspiratory effort, the MP during noninvasive respiratory support has been suggested as one of the most comprehensive variables to assess lung derangements (32); it has been computed taking into account the ΔPes, the respiratory rate, and the tidal volume. The MP during noninvasive respiratory support arises from the combination of the energy generated by the respiratory muscles and the energy delivered by respiratory support device, and it is associated to the clinical outcome (32). In our study, the MP was significantly reduced by the application of HFNC and helmet CPAP at 5 cm H_2_O of PEEP; on the contrary, the application of 10 cm H_2_O of PEEP worsened the MP without additional benefits in terms of respiratory mechanics or inspiratory effort and with only a modest increase in oxygenation.

### Gas Exchange

Both HFNC and helmet CPAP reduced VR, without any difference in Paco_2_ compared with COT (16, 17).

Concerning arterial oxygenation, it was not different among HFNC and helmet CPAP with a similar Fio_2_, but significantly higher compared with COT. Similarly to the improvement of Co_2_ clearance, the increase in oxygenation could be due to the PEEP effect with a better ventilation/perfusion distribution with both noninvasive ventilatory supports. It was previously shown that the application of HFNC in healthy subjects with a gas flow rate between 40 and 60 L/min pressurized the upper airways up to 3–7 cm H_2_O (13, 47).

Furthermore, there was a positive linear relationship between the flow delivered by the HFNC and the generated pressure (15). For an average 10 L/min increase in gas flow, the mean Paw increased by 1.2 cm H_2_O; in addition, as the gas flow increased, this was associated with an increase in the EELV (14). According to these data, the gas flow rate during HFNC was maintained between 40 and 70 L/min.

The increase in PEEP up to 10 cm H_2_O with helmet CPAP did not further ameliorate the arterial oxygenation or minute ventilation; on the contrary, the Paco_2_ was significantly higher mainly due to the lung overdistension with an increase in the alveolar dead space.

### Strengths and Limitations

Strength of this study is the contemporaneous measurements of Pes and minute ventilation, which allowed the computation of the indices of inspiratory effort and MP during helmet CPAP and HFNC. Limitations include the small sample size, the prevalence of mild and moderate AHRF patients and the duration of each step of the study protocol, that is, 20 minutes, along with the absence of washout periods among different respiratory supports, which could have resulted in a carry-over effect.

## CONCLUSIONS

In patients with mild and moderate AHRF, the application of HFNC and helmet CPAP with 5 cm H_2_O ameliorated respiratory mechanics, reduced inspiratory effort, and increased oxygenation with respect to conventional oxygen support with an oxygen mask; the application of 10 cm H_2_O of PEEP during helmet CPAP did not guaranteed additional benefits in terms of inspiratory effort or oxygenation, but worsened MP.

## Supplementary Material
