# Supplementary material for: Effects of High-Flow Nasal Cannula and Helmet Continuous Positive Airway Pressure in Acute Hypoxemic Respiratory Failure
Source: Crit Care Med. 2026 Apr 1;54(6):1420–30. doi: 10.1097/CCM.0000000000007116 (PMC13185722; doi:10.1097/CCM.0000000000007116)
Supplement: Supplementary file 1 [file ccm-54-1420-s001.docx]

**EFFECTS OF HIGH FLOW NASAL CANNULA AND HELMET CPAP IN ACUTE HYPOXEMIC RESPIRATORY FAILURE**

**Supplementary Digital Content**

Silvia Coppola MD^1^, Mariarosa Pelliccia MD^1^, Tommaso Pozzi^1,2^, Giulia Catozzi MD^2^, Cosmo Rocco MD^2^, Alessandro Monte MD^2^, Geraldina Besana^1,2^ MD and Davide Chiumello MD^1,2,3^

^1^ Department of Anesthesia and Intensive Care, ASST Santi Paolo e Carlo, San Paolo University Hospital Milan, Italy

^2^ Department of Health Sciences, University of Milan, Italy

^3^ Coordinated Research Center on Respiratory Failure, University of Milan, Italy

**Additional Material and Methods**

*Esophageal pressure measurement by the esophageal catheter*

Esophageal pressure was measured by using a radiopaque catheter equipped with a balloon in the lower part (Nutrivent, Sidam Srl., Modena, Italy). Before the insertion the esophageal catheter was emptied of air and closed with a three-way stopcock. Then, it was introduced trans-orally and advanced to reach the stomach (generally at a least depth of 55 cm from the mouth). The balloon was inflated with an air volume of 4 mL. The intragastric position of the catheter was confirmed by a rise in intra-abdominal pressure following external manual epigastric compression. Finally, it was retracted into the esophagus (*i.e.*, confirmed by the presence of cardiac artifacts in the pressure tracing), at a distance between 40-45 cm from the mouth.

*Tidal volume and respiratory rate measurement by ExSpiron*

The non-invasive respiratory monitoring system (ExSpiron 1Xi, Senzime, Uppsala, Sweden) measures bioelectrical impedance, which is the tissue’s opposition to carrying an alternating electrical current. Impedance measurements correlate with the volume of air in the lungs has been used. Software algorithms calculate minute ventilation, tidal volume and respiratory rate without the need to calibrate. The respiratory parameters displayed on the monitoring screen of the ExSpiron 1Xi represent a 30 second moving average of the indicated metrics. A previous study comparing the agreement between ExSpiron non-invasive respiraotory monitoring system measurements in sedated and intubated patients showed a good accuracy, with an average tidal volume difference of 40 ml (bias 0.4%, precision 7.3%, accuracy 9.1%) and a the average respiratory difference was -0.22 bpm (bias -1.8%, precsion 3.7%, accuracy 4.1%) (1). A similar accuracy was also found in not intubated setting and in supine and prone position (2, 3).

The electrode PadSet were placed on the thorax positioned at the sternal notch, xiphoid and in the right mid axillary line at the level of xiphoid; they were maintained in the original position for the entire duration of the study. Respiratory measurements were taken at the end of each phase of respiratory support, after checking the stability of the signal for two minutes.

1. Voscopoulos CJ, MacNabb CM, Brayanov J, et al.: The evaluation of a non-invasive respiratory volume monitor in surgical patients undergoing elective surgery with general anesthesia. *J Clin Monit Comput* 2015; 29:223–230

2. Voscopoulos C, Brayanov J, Ladd D, et al.: Evaluation of a Novel Noninvasive Respiration Monitor Providing Continuous Measurement of Minute Ventilation in Ambulatory Subjects in a Variety of Clinical Scenarios. *Anesth Analg* 2013; 117:91–100

3. Pozzi T, Coppola S, Chiodaroli E, et al.: The evaluation of a non-invasive respiratory monitor in ards patients in supine and prone position. *J Clin Monit Comput* 2024; 38:671–677
